# Supplementary material for: INPP5E regulates CD3ζ enrichment at the immune synapse by phosphoinositide distribution control
Source: Commun Biol. 2023 Sep 5;6:911. doi: 10.1038/s42003-023-05269-0 (PMC10480498; doi:10.1038/s42003-023-05269-0)
Supplement: Supplementary file 1 — Description of Additional Supplementary Files [file 42003_2023_5269_MOESM1_ESM.pdf]

Description of Additional Supplementary Files

File name: Supplementary Data 1
Description: The source data behind the graphs in the main figures.

File name: Supplementary Data 2
Description: The source data behind the graphs in the supplementary figures.
