# Supplementary material for: INPP5E regulates CD3ζ enrichment at the immune synapse by phosphoinositide distribution control
Source: Commun Biol. 2023 Sep 5;6:911. doi: 10.1038/s42003-023-05269-0 (PMC10480498; doi:10.1038/s42003-023-05269-0)
Supplement: Supplementary file 2 — Supplementary figures [file 42003_2023_5269_MOESM2_ESM.pdf]

Supplementary figures

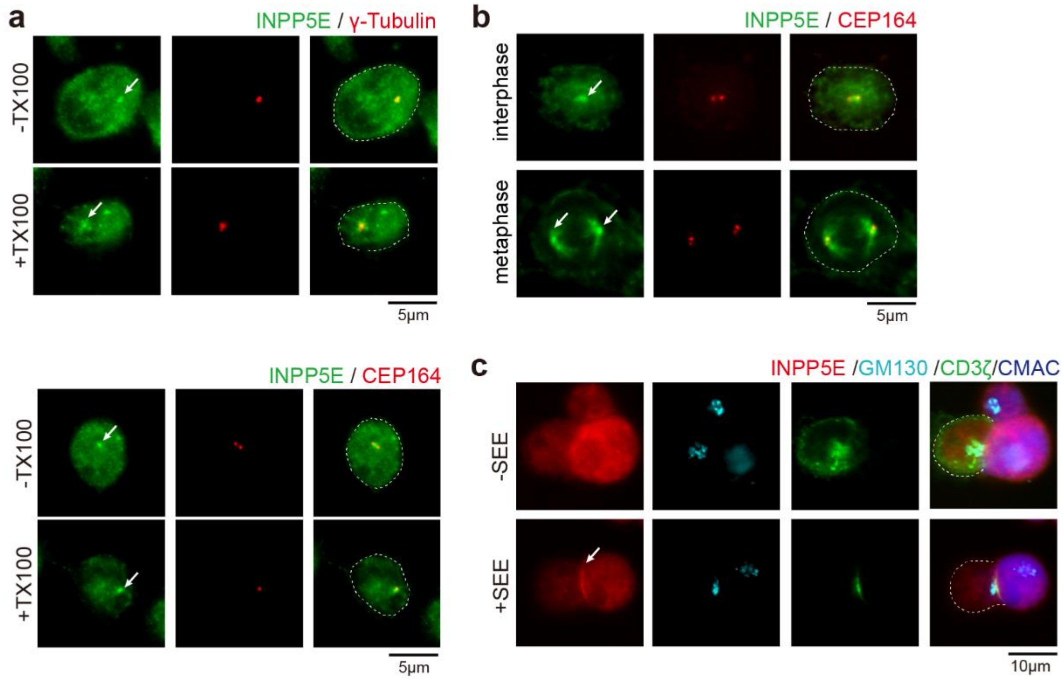

Supplementary Fig. 1: Localization of centrosome and cilia-associated proteins in resting and activated T cells.

a Immunostaining of INPP5E in Jurkat cells, either without or with Triton X-100 extraction before fixation. Cells were co-stained with anti-γ-tubulin or anti-CEP164 antibodies as centriole markers. Scale bar, 5 µm. **b** Immunostaining of INPP5E in Jurkat cells in interphase (upper) and metaphase (lower). Scale bar, 5 µm. **c** Immunostaining of INPP5E in conjugates of Jurkat T cells (Left) and CMAC-labeled Raji B cells (Right), in the presence (+) or absence (–) of SEE. Cells were co-stained with an anti-GM130 antibody as the Golgi marker. Scale bar, 10 µm.

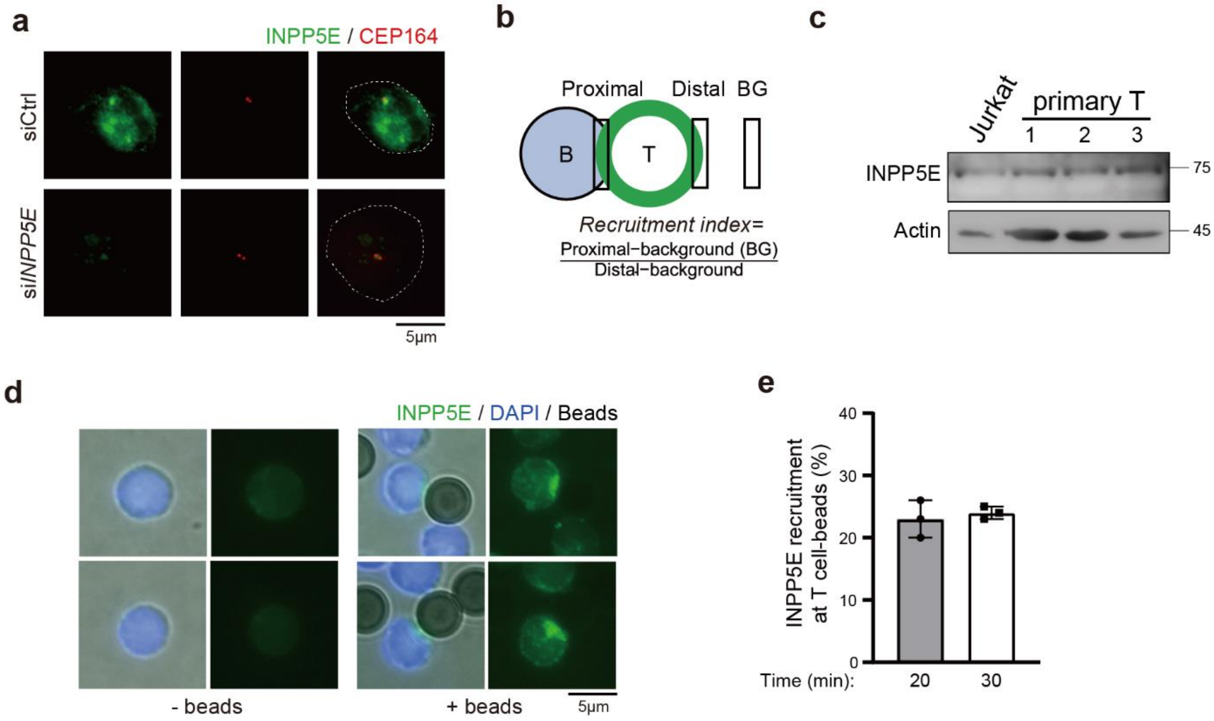

Supplementary Fig. 2: Knockdown INPP5E in Jurkat cells.

a Immunostaining of INPP5E in siCtrl or si*INPP5E* transfected Jurkat cells. Cells were co-stained with an anti-CEP164 antibody. Scale bar, 5 µm. **b** The calculation method of the recruitment index is shown. **c** Cell lysates from Jurkat and T cells from three healthy donors were resolved by SDS-PAGE and immunoblots with anti-INPP5E antibody. Actin was used as the internal control. **d** Immunostaining of INPP5E in conjugates of primary mouse pan T cells and anti-CD3/CD28 coated beads. Cells were co-stained with DAPI. Scale bar, 5 µm. **e** The recruitment of INPP5E at T cell-beads from (**d**) was quantified. N=3 independent experiments.

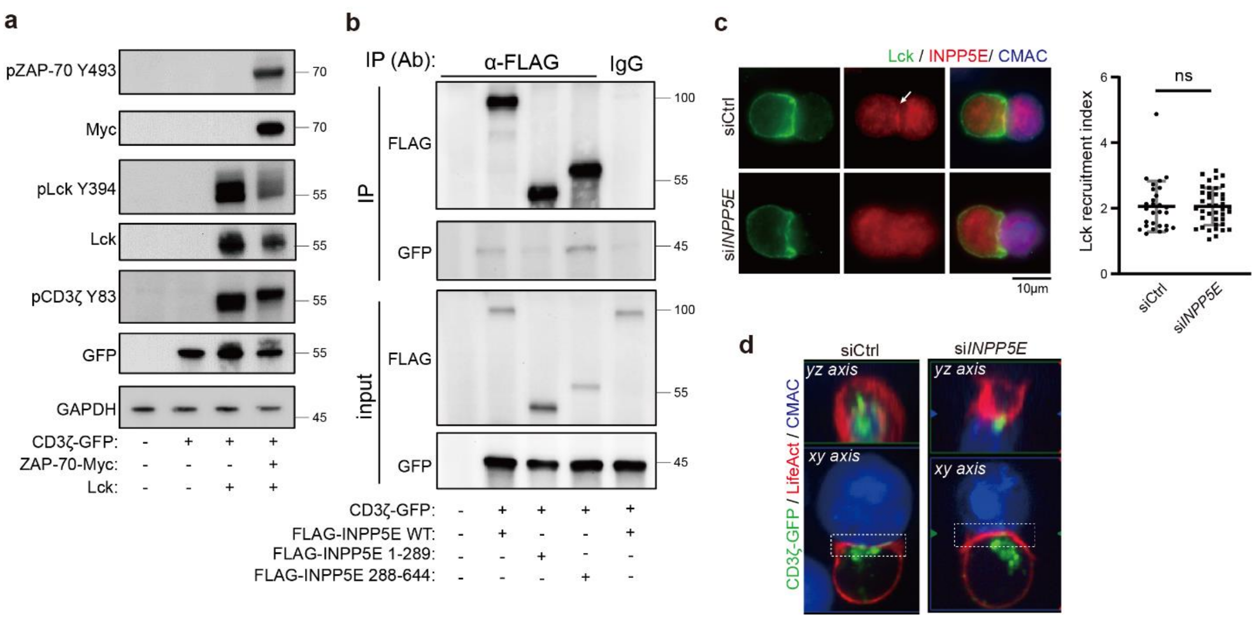

Supplementary Fig. 3: INPP5E utilizes different domains for immune synapse docking and CD3ζ interaction.

a HEK293T cells were transfected with listed constructs. Cell lysates were resolved by SDS-PAGE and immunoblotted with indicated antibodies. **b** HEK293T cells were co-expressed with CD3ζ-GFP and INPP5E deletion mutant. Cells were then lysed and immunoprecipitated (IP) with an anti-Flag antibody. The immuno-precipitates were resolved by SDS-PAGE and immunoblotted with indicated antibodies. "IP Ab" indicates the antibodies used for IP in each lane. The IgG lane was used for non-specific antibody control. Images are representative of at least three experiments. **c** Jurkat cells were transfected with either siCtrl or si*INPP5E.* Immunostaining of Lck and INPP5E in conjugates of Jurkat T cells and CMAC-labeled SEE-pulsed APCs. Quantification of Lck recruitment index in conjugates was from two independent experiments. n = 28 conjugates for siCtrl, and n = 40 conjugates for si*INPP5E*. Scale bar = 10 μm. Unpaired student T-test. Arrows indicate localization of INPP5E. ns, not significant. **d** Representative 3D-reconstructed *en face* at immune synapse in the yz plane after 5 min conjugation from Fig. 4h. Dotted white squares indicate the regions that were selected for 3D reconstruction.

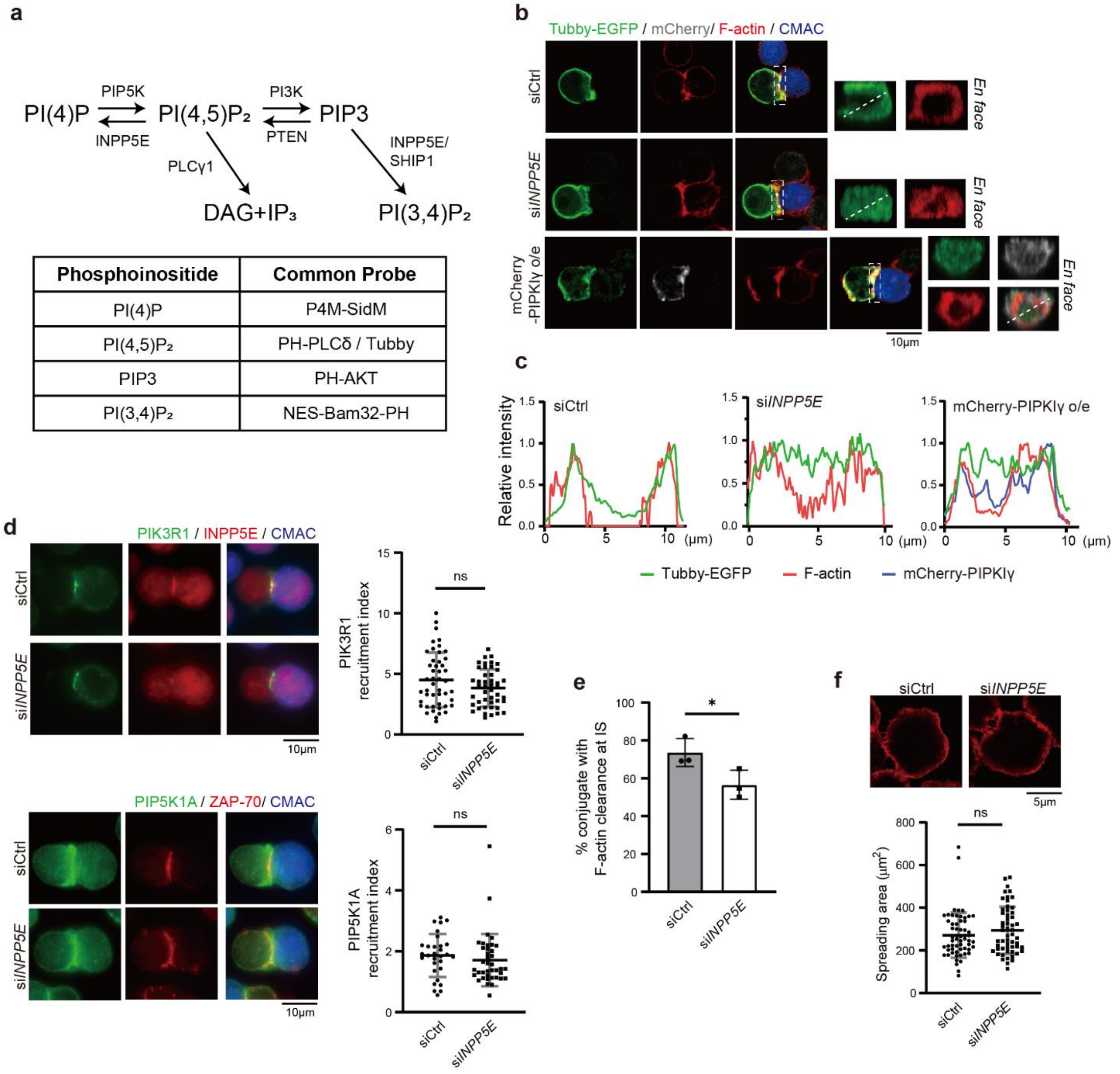

Supplementary Fig. 4: INPP5E modulates PI(4,5)P_2 environment at the immune synapse.

a Summary of INPP5E related phosphoinositides and common fluorescence probes for detecting phosphoinostides. **b, c** Immunostaining of Tubby-GFP in conjugates of Jurkat T cells and CMAC-labeled SEE-pulsed APCs. In **b**, images are shown in the xy plane (scale bar, 10 µm) or 1.0 µm 3D-reconstructed *en face* at the T cell-APC contact site in the xz plane (scale bar, 3 µm). Cells were stained with an anti-GFP antibody. Alexa Fluor 647 phalloidin was stained to visualize F-actin. Dotted white squares indicate the regions that were selected for 3D reconstruction. Upper, control siRNA transfected cells; middle, INPP5E-specific siRNA transfected cells; lower, mCherry-PIPKIγ overexpressed (o/e) cells. Images are representative of two experiments. In **c**,

line profiling (derived from the white lines in the merged *en face* images) shows the fluorescence intensities of F-actin, Tubby-GFP, and mCherry-PIPKIγ. **d** Jurkat cells were transfected with either control or *INPP5E*-specific siRNA. Immunostaining of PIK3R1 and INPP5E (upper), PIP5K1A and ZAP-70 (lower) in conjugates of Jurkat T cells and CMAC-labeled SEE-pulsed APCs. Quantification of PIK3R1 and PIP5K1A recruitment index in conjugates was from two independent experiments. For PIK3R1, n = 27 conjugates for siCtrl, and n = 30 conjugates for si*INPP5E*. For PIP5K1A, n = 35 conjugates for siCtrl, and n = 31 conjugates for si*INPP5E*. Scale bar = 10 μm. Arrows indicate localization of INPP5E. **e** Quantification of F-actin clearance at the immune synapse in conjugates. n = 54 conjugates for siCtrl, and n = 74 conjugates for *INPP5E* knockdown cells. Unpaired t-test. *P < 0.05. **f** Jurkat cells were transfected with either control or INPP5E-specific siRNA. Immunostaining of F-actin in spreading Jurkat cells on anti-CD3/CD28-coated coverslips for 10 min and analyzed by confocal microscopy. n = 59 cells for siCtrl and n = 55 cells for si*INPP5E*. Scale bar = 5 μm. Unpaired student t-test were used for quantification for this figure. Error bars indicate mean ± SD. ns, not significant. *P < 0.05. Images are representative of three experiments.

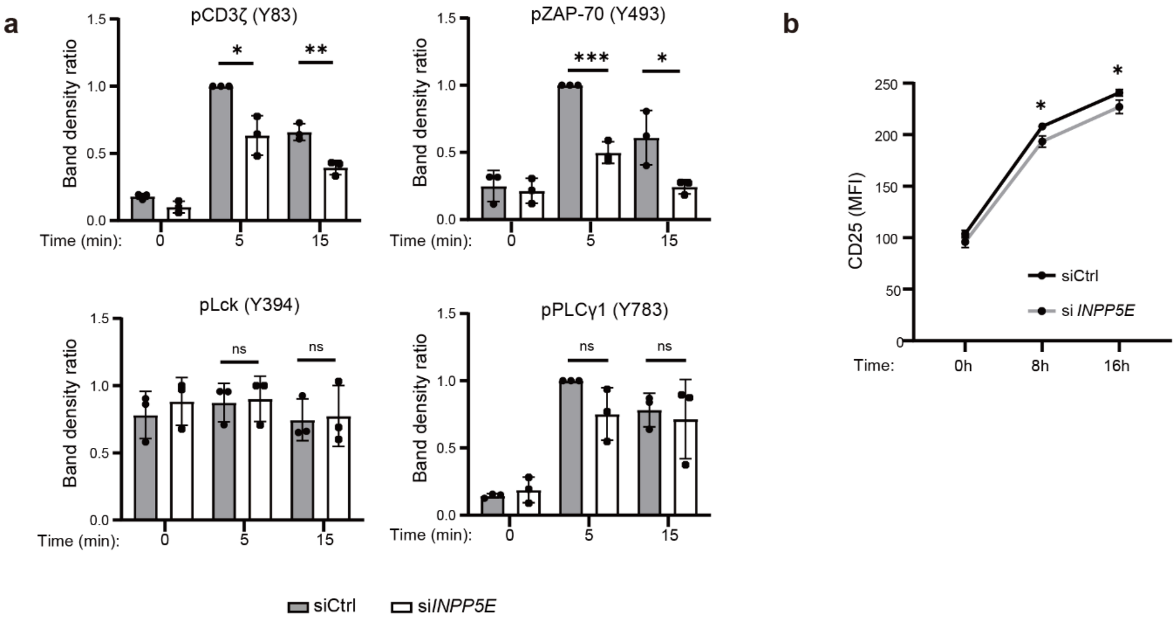

Supplementary Fig. 5: INPP5E is required for efficient proximal TCR signaling and effector functions.

a The phosphorylation levels of indicated proteins upon T cell activation were quantified. The results were an average of three independent assays and were all normalized to non-phosphorylated proteins. **b** Jurkat cells were transfected with either control or *INPP5E*-specific siRNA. The surface expression of CD25 in siCtrl and si*INPP5E* cells were analyzed by FACS. The results were an average of three independent assays. Unpaired student t-test were used for quantification for this figure. Error bars indicate mean ± SD. *P < 0.05. **P <0.01. ***P <0.001. ns, not significant.

Fig. 2a

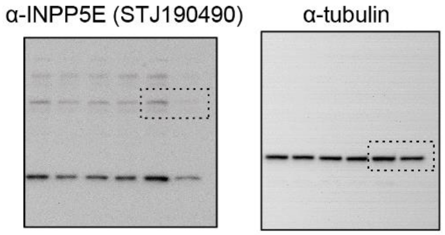

Fig. 3c

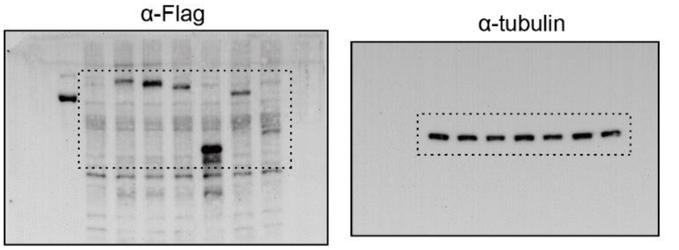

Fig. 4a

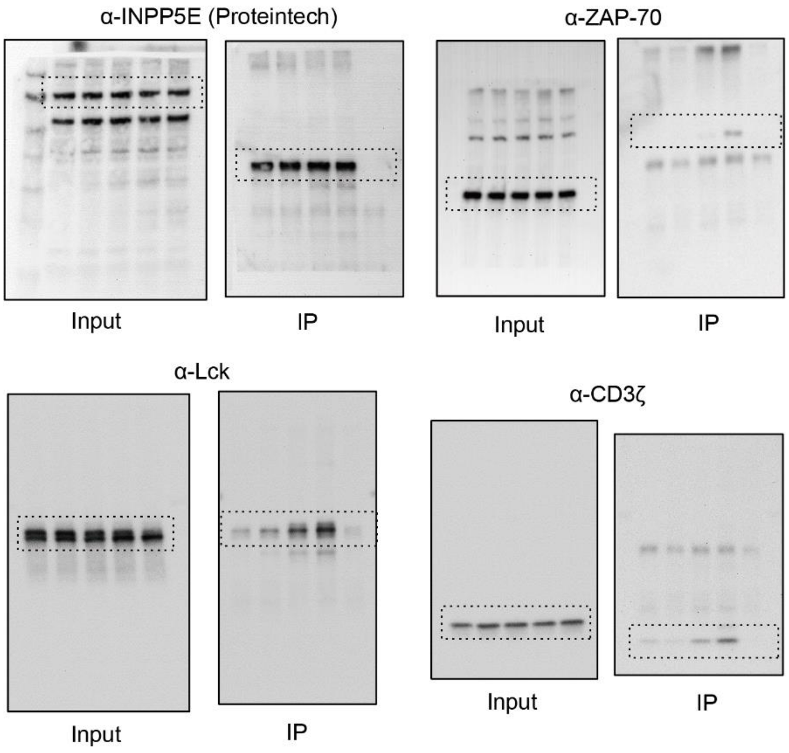

Fig. 4b

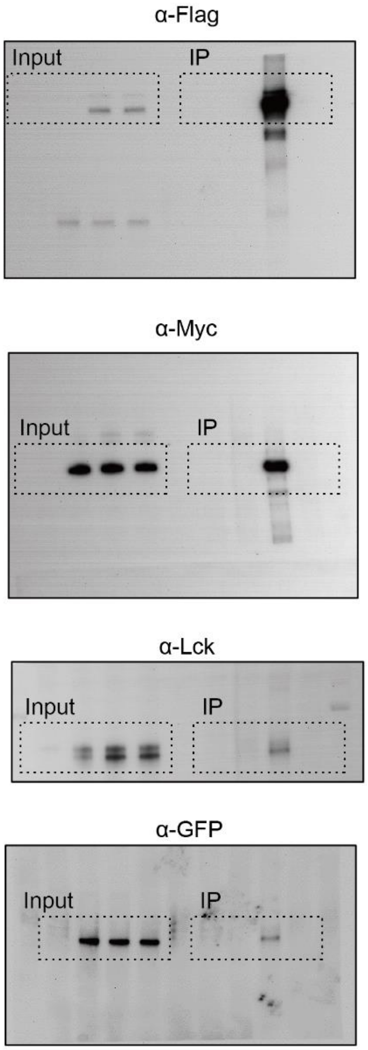

Fig. 4c

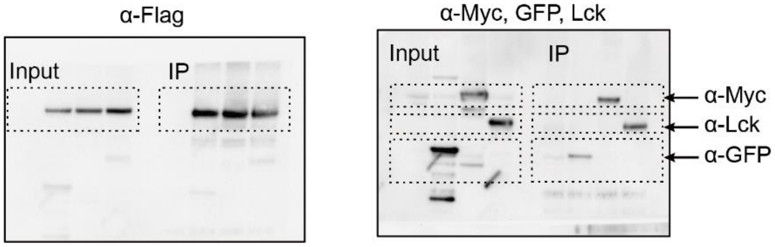

Fig. 6a

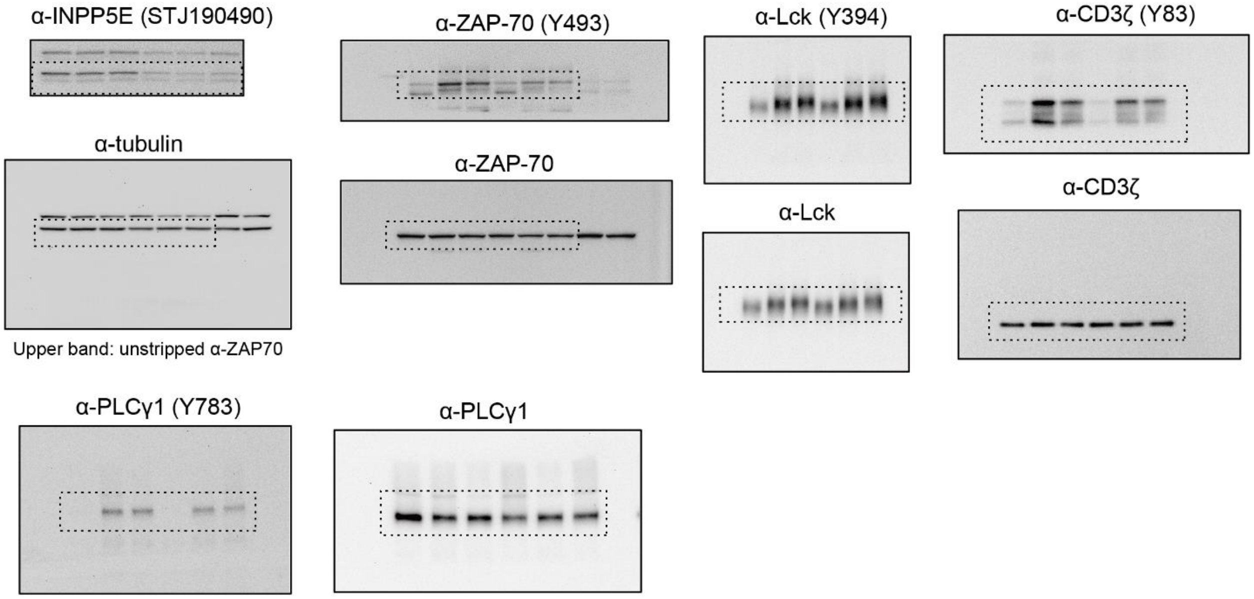

Fig. 6d

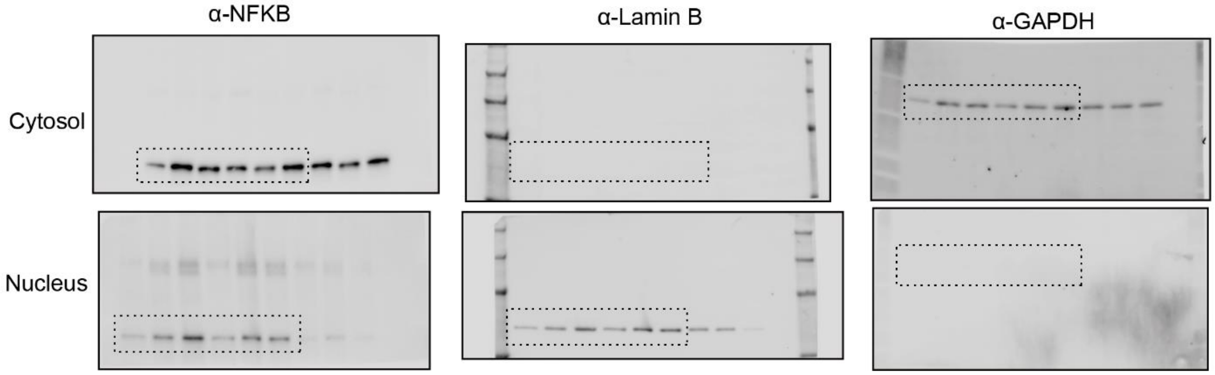

Supplementary Fig. 6: uncropped western blots of main figures.

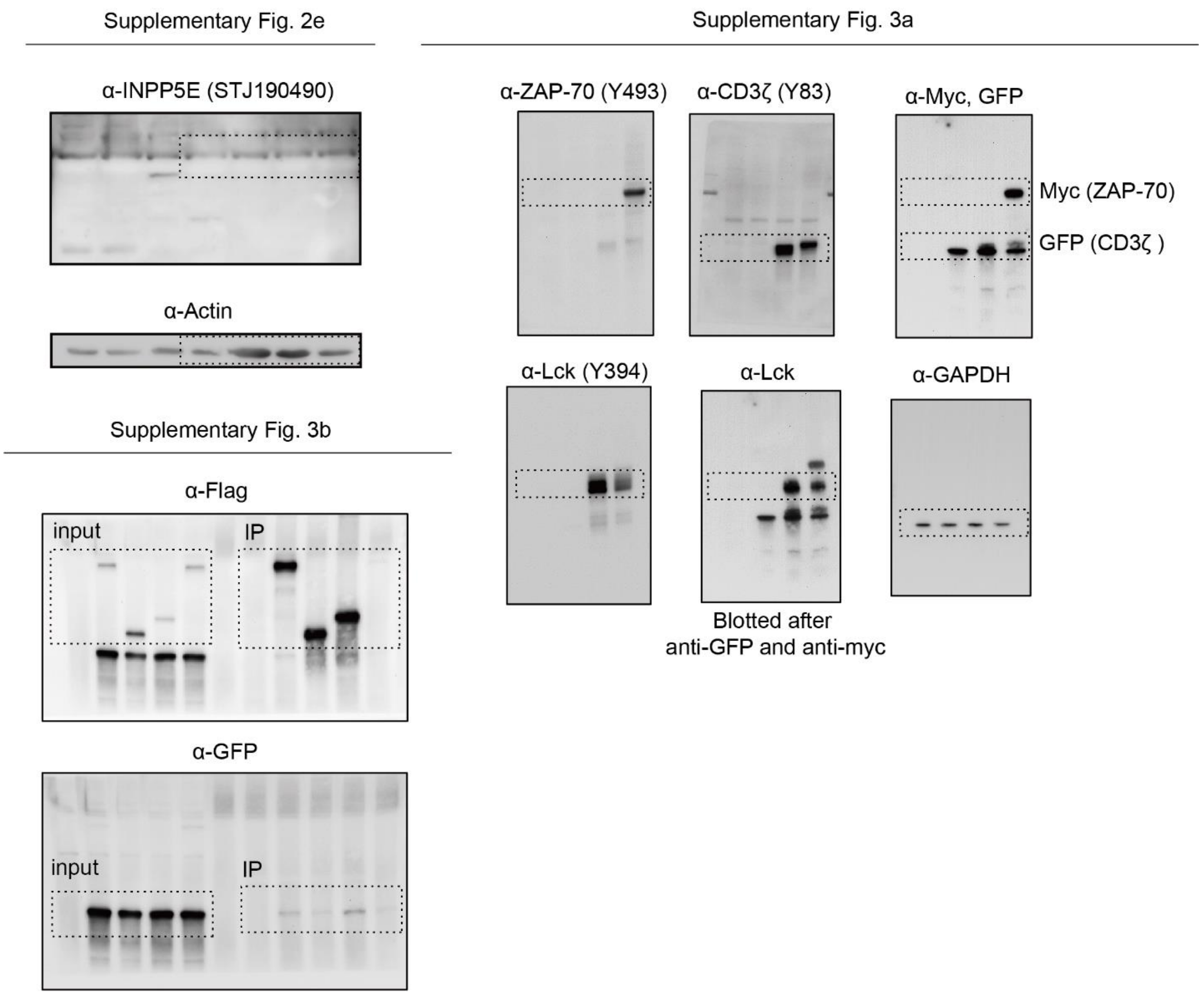

Supplementary Fig. 7: uncropped western blots of Supplementary Figures.

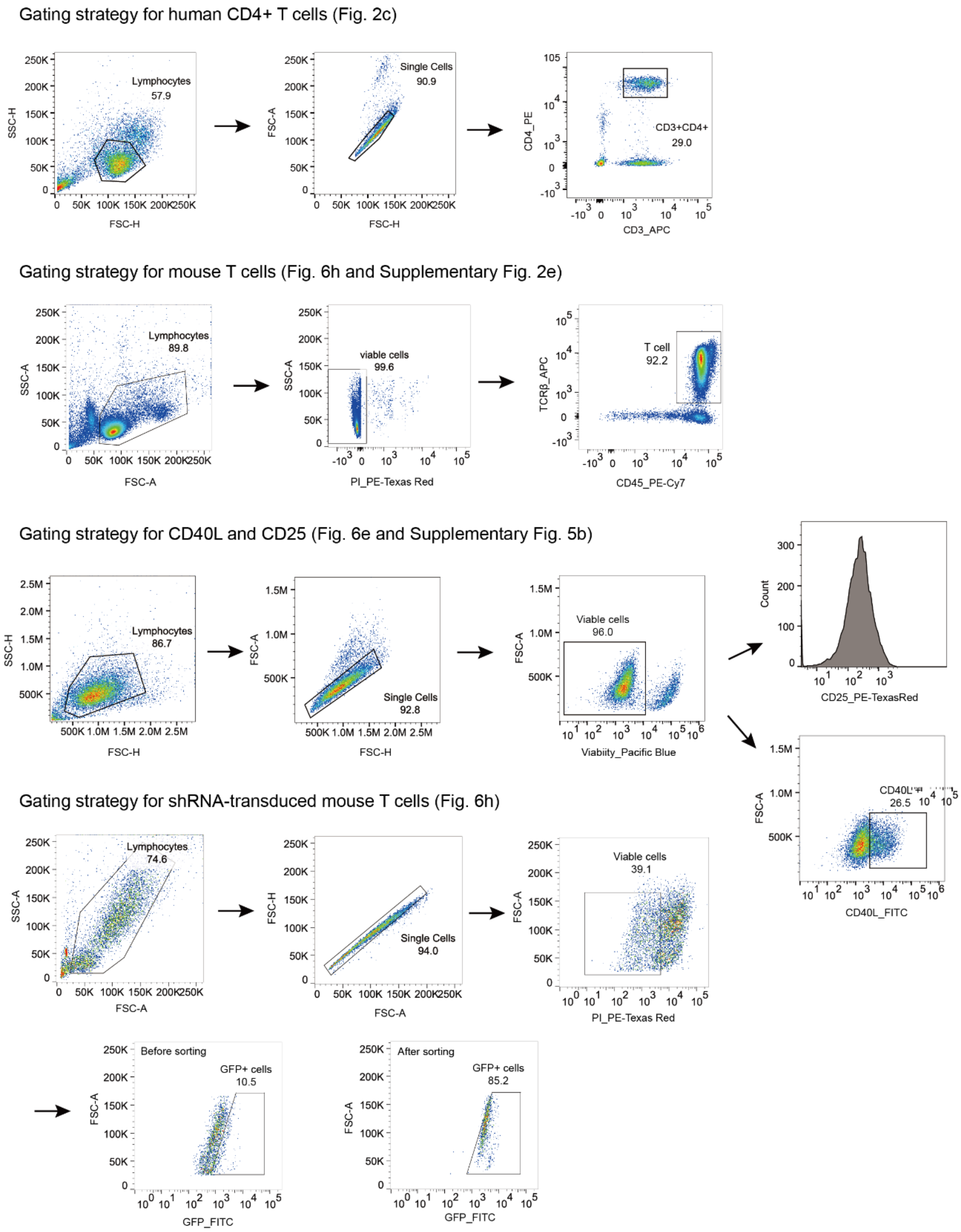

Supplementary Fig. 8: Gating strategy for flow cytometry analysis.
