# Supplementary material for: INPP5E regulates CD3ζ enrichment at the immune synapse by phosphoinositide distribution control
Source: Commun Biol. 2023 Sep 5;6:911. doi: 10.1038/s42003-023-05269-0 (PMC10480498; doi:10.1038/s42003-023-05269-0)
Supplement: Supplementary file 5 — Reporting Summary [file 42003_2023_5269_MOESM5_ESM.pdf]

nature portfolio

Corresponding author(s): Won-Jing Wang & Jung-Chi Liao

Last updated by author(s): Aug 13, 2023

Reporting Summary

Nature Portfolio wishes to improve the reproducibility of the work that we publish. This form provides structure for consistency and transparency in reporting. For further information on Nature Portfolio policies, see our Editorial Policies and the Editorial Policy Checklist.

Statistics

For all statistical analyses, confirm that the following items are present in the figure legend, table legend, main text, or Methods section.

n/a	Confirmed	
☐	☒	The exact sample size (*n*) for each experimental group/condition, given as a discrete number and unit of measurement
☐	☒	A statement on whether measurements were taken from distinct samples or whether the same sample was measured repeatedly
☐	☒	The statistical test(s) used AND whether they are one- or two-sided<br>*Only common tests should be described solely by name; describe more complex techniques in the Methods section.*
☐	☒	A description of all covariates tested
☐	☒	A description of any assumptions or corrections, such as tests of normality and adjustment for multiple comparisons
☐	☒	A full description of the statistical parameters including central tendency (e.g. means) or other basic estimates (e.g. regression coefficient) AND variation (e.g. standard deviation) or associated estimates of uncertainty (e.g. confidence intervals)
☐	☒	For null hypothesis testing, the test statistic (e.g. *F*, *t*, *r*) with confidence intervals, effect sizes, degrees of freedom and *P* value noted<br>*Give P values as exact values whenever suitable.*
☒	☐	For Bayesian analysis, information on the choice of priors and Markov chain Monte Carlo settings
☒	☐	For hierarchical and complex designs, identification of the appropriate level for tests and full reporting of outcomes
☐	☒	Estimates of effect sizes (e.g. Cohen's *d*, Pearson's *r*), indicating how they were calculated

Our web collection on statistics for biologists contains articles on many of the points above.

Software and code

Policy information about availability of computer code

Data collection	All software used in this studies are commercial available. FACS data was collected using NovoCyte Analyzers and NovoExpress software(v1.2.5) from ACEA Bioscience. Inc.. Cell sorting was collected using BD FACSAria™ cell sorter ; RT-qPCR data was collected using StepOnePlus™ Real-Time PCR System (Thermo Fisher Scientific); Microscopy images were collected with Carl Zeiss AG, ZEN 2.3 or Molecular Devices MetaMorph 7.10.3. No other custom codes were used in this study.
Data analysis	Carl Zeiss AG, ZEN 2.3, Image J 1.5, and Imaris 8.3 were used to analyze images from microscopes. Flow cytometry results were analyzed with FlowJo 10.0. Data graphs for ELISA, image quantification were prepared in GraphPad Prism v8 or Excel 2016.

For manuscripts utilizing custom algorithms or software that are central to the research but not yet described in published literature, software must be made available to editors and reviewers. We strongly encourage code deposition in a community repository (e.g. GitHub). See the Nature Portfolio guidelines for submitting code & software for further information.

Data

Policy information about availability of data

All manuscripts must include a data availability statement. This statement should provide the following information, where applicable:

- Accession codes, unique identifiers, or web links for publicly available datasets
- A description of any restrictions on data availability
- For clinical datasets or third party data, please ensure that the statement adheres to our policy

The data that support the findings in this work are available within the paper and Supplementary Information (Supplementary text and Supplementary Figure 1-5). Uncropped, full western blot images and gels are provided in Supplementary Figure 7. Source data are provided with this paper.

Human research participants

Policy information about studies involving human research participants and Sex and Gender in Research.

Reporting on sex and gender	All other studies with non-diseased primary human cells were performed on samples obtained from 201706119RIND following protocols approved by Institutional Review Board. No potential self-selection bias or other biases that could impact results are presented, no sex- and gender-based analyses have been performed.
Population characteristics	Primary human peripheral blood mononuclear cells (PBMCs) were obtained from three healthy donors with no known medical conditions. Blood from healthy donors were obtained after informed consent.
Recruitment	Healthy volunteers were recruited at National Taiwan University Hospital. The overview of the research project was provided orally or through recruitment flyers.
Ethics oversight	Institutional Review Board (IRB) of National Taiwan University Hospital.

Note that full information on the approval of the study protocol must also be provided in the manuscript.

Field-specific reporting

Please select the one below that is the best fit for your research. If you are not sure, read the appropriate sections before making your selection.

☒ Life sciences ☐ Behavioural & social sciences ☐ Ecological, evolutionary & environmental sciences

For a reference copy of the document with all sections, see nature.com/documents/nr-reporting-summary-flat.pdf

Life sciences study design

All studies must disclose on these points even when the disclosure is negative.

Sample size	Sample size was determined empirically, based on exploratory experiments, based on published literature with similar methodology. The sample sizes were considered sufficient due to the large effect sizes which allowed the biological interpretation of the results obtained.
Data exclusions	No data were excluded from the study.
Replication	Reported experiments were repeated at least 2 times with comparable results.
Randomization	Samples were not randomized for this study since no suggestive rating of data was involved.
Blinding	Blinding was not relevant for this study since no suggestive rating of data was involved.

Reporting for specific materials, systems and methods

We require information from authors about some types of materials, experimental systems and methods used in many studies. Here, indicate whether each material, system or method listed is relevant to your study. If you are not sure if a list item applies to your research, read the appropriate section before selecting a response.

Materials & experimental systems

n/a	Involved in the study
	☒ Antibodies
	☒ Eukaryotic cell lines
☒	Palaeontology and archaeology
	☒ Animals and other organisms
☒	Clinical data
☒	Dual use research of concern

Methods

n/a	Involved in the study
☒	ChIP-seq
	☒ Flow cytometry
☒	MRI-based neuroimaging

Antibodies

Antibodies used

The indicated antibodies used for immunoblotting (IB) and immunofluorescence (IF) in this study include: FLAG M2 (F1804; 1:500 IF) and γ-tubulin (T6557; 1:500 IF) from Sigma-Aldrich; INPP5E (STJ190490 ; 1:500 IB), pCD3ζY83 (ab68236; 1:1000 IB, 1:200 IF), and CEP290 (ab84870; 1:200 IF) from Abcam; CD3 (OKT3, 317302, 1:500 IF) from BioLegend; CD3ζ (sc-1239; 1:2000 IB, 1:500 IF), α-tubulin (sc-32293; 1:10000 IB), GM130 (sc-16268; 1:500 IF), GAPDH (sc-365062; 1:2000 IB) and normal rabbit IgG (sc-2027) from Santa Cruz; NF-κB (#8242; 1:2000 IB), pZAP-70Y493 (#2704; 1:1000 IB, 1:200 IF), PLCγ1 (#5690,1:1000 IB), pPLCγ1Y783 (#2821, 1:1000 IB), Lck (#2984, 1:1000 IB), LckY505 (#2751, 1:1000 IB), and SrcY416 (#6943, 1:2000 IB) from Cell Signaling Technology; ZAP-70 (1X17371; 1:500 IB) from Genetex; Myc-Tag (AE009; 1:10000 IB) from ABclonal; Centrin (04-1624; 1:500 IF) from Millipore; mCherry (PA5-34974, 1:1000 IF), GFP (A6455; 1:1000 IF, 1:10000 IB), and DYKDDDDK (PA1-984B; 1:2000 IB) from Invitrogen; Lamin B1 (66095-1-Ig; 1:2000 IB), CEP97 (22050-1-AP; 1:200 IF), CEP164 (22227-1-AP; 1:200 IF), RPGRIP1L (55160-1-AP; 1:200 IF), ARL13B (17711-1-AP; 1:200 IF), and INPP5E (17797-1-AP, 1:200 IF) from Proteintech. CEP164 (1F3G10, 1:2000 IF) was kindly provided by C. Morrison (National University of Ireland, Galway). For T cell activation, anti-CD3 (OKT3, 317325, BioLegend) and anti-CD28 (cd28.2, 302902, BioLegend) were used. For flow cytometry, PE-CF594 anti-CD25 (562403, BD bioscience) and AF488 anti-CD40L (310815, Biolegend) were used.

Validation

Specificity of INPP5E antibodies (STJ190490 , St John's labortory) was validated by western blot in INPP5E knockdown cells . All other antibodies were bought from commercial vendors and validation for indicated species and applications can be found on the manufacturers website or the provided scientific citations on the same website.

Eukaryotic cell lines

Policy information about cell lines and Sex and Gender in Research

Cell line source(s)

HEK293T, Jurkat, Clone E6-1 and Raji cells were purchased from BCRC (#60424 and #60116; Bioresource Collection and Research Center, Hsinchu, Taiwan). 293FT cells were purchased from Invitrogen.

Authentication

All cell lines used in this study were authenticated by STR profiling.

Mycoplasma contamination

Cell lines used were tested negatively for mycoplasma contamination.

Commonly misidentified lines
(See ICLAC register)

None of commonly misidentified cell lines were used in this study.

Animals and other research organisms

Policy information about studies involving animals; ARRIVE guidelines recommended for reporting animal research, and Sex and Gender in Research

Laboratory animals

Both male and female C57BL/6J mice, aged 8-10 weeks, were originally purchased from the National Laboratory Animal Center and maintained in the specific-pathogen-free (SPF) facility at the animal center of National Yang Ming Chiao Tung University (NYCU).

Wild animals

This study did not involve wild animals.

Reporting on sex

Experiments were performed either with male or female mice.

Field-collected samples

This study did not involve field-collected samples.

Ethics oversight

All experimental procedures of animal studies were approved and performed in accordance with the Institutional Animal Care and Use Committee guidelines of NYCU (IACUC #1090115).

Note that full information on the approval of the study protocol must also be provided in the manuscript.

Flow Cytometry

Plots

Confirm that:

☒ The axis labels state the marker and fluorochrome used (e.g. CD4-FITC).

☒ The axis scales are clearly visible. Include numbers along axes only for bottom left plot of group (a 'group' is an analysis of identical markers).

☒ All plots are contour plots with outliers or pseudocolor plots.

☒ A numerical value for number of cells or percentage (with statistics) is provided.

Methodology

Sample preparation	Jurkat cells were collected, washed with PBS and stained with antibodies.
Instrument	NovoCyte Analyzers (ACEA Biosciences, Inc)
Software	BD FACS Diva 6 was used to distinguish populations. MicroSoft Excel (v2212) and GraphPad Prism (v9.3.0) were used to visualize and perform statistical analysis.
Cell population abundance	At least 10,000 events were analyzed per replicate.
Gating strategy	Lymphocyte population was identified using FSC-A/SSC-A gating. From the lymphocyte population, single cells were identified using FSC-A and H gates. From the single cell population, live cells were characterized using the Zombie Violet™ Fixable Viability dye. Downstream activation markers (CD40L, CD25) were gated on live cells.

☒ Tick this box to confirm that a figure exemplifying the gating strategy is provided in the Supplementary Information.
